# Fukutin is prerequisite to ameliorate muscular dystrophic phenotype by myofiber-selective LARGE expression

**DOI:** 10.1038/srep08316

**Published:** 2015-02-09

**Authors:** Yoshihisa Ohtsuka, Motoi Kanagawa, Chih-Chieh Yu, Chiyomi Ito, Tomoko Chiyo, Kazuhiro Kobayashi, Takashi Okada, Shin'ichi Takeda, Tatsushi Toda

**Affiliations:** 1 Division of Neurology/Molecular Brain Science, Kobe University Graduate School of Medicine, Kobe, 650-0017, Japan; 2 Department of Molecular Therapy, National Institute of Neuroscience, National Center of Neurology and Psychiatry, Kodaira, 187-8502, Japan

## Abstract

α-Dystroglycanopathy (α-DGP) is a group of muscular dystrophy characterized by abnormal glycosylation of α-dystroglycan (α-DG), including Fukuyama congenital muscular dystrophy (FCMD), muscle-eye-brain disease, Walker-Warburg syndrome, and congenital muscular dystrophy type 1D (MDC1D), etc. LARGE, the causative gene for MDC1D, encodes a glycosyltransferase to form [-3Xyl-α1,3GlcAβ1-] polymer in the terminal end of the post-phosphoryl moiety, which is essential for α-DG function. It has been proposed that LARGE possesses the great potential to rescue glycosylation defects in α-DGPs regardless of causative genes. However, the in vivo therapeutic benefit of using LARGE activity is controversial. To explore the conditions needed for successful LARGE gene therapy, here we used *Large*-deficient and *fukutin*-deficient mouse models for MDC1D and FCMD, respectively. Myofibre-selective LARGE expression via systemic adeno-associated viral gene transfer ameliorated dystrophic pathology of *Large*-deficient mice even when intervention occurred after disease manifestation. However, the same strategy failed to ameliorate the dystrophic phenotype of *fukutin*-conditional knockout mice. Furthermore, forced expression of *Large* in *fukutin*-deficient embryonic stem cells also failed to recover α-DG glycosylation, however coexpression with *fukutin* strongly enhanced α-DG glycosylation. Together, our data demonstrated that fukutin is required for LARGE-dependent rescue of α-DG glycosylation, and thus suggesting new directions for LARGE-utilizing therapy targeted to myofibres.

α-Dystroglycanopathy (α-DGP) is a genetically and clinically heterogeneous group of muscular dystrophy^1^,^2^ for which more than 15 causative genes have been identified^3^,^4^,^5^,^6^,^7^,^8^,^9^,^10^,^11^,^12^,^13^,^14^,^15^,^16^,^17^,^18^,^19^,^20^,^21^: *POMT1, POMT2, POMGnT1, fukutin, FKRP, LARGE, ISPD, GTDC2* (*POMGnT2*), *DAG1, TMEM5, B3GALNT2, SGK196* (*POMK*), *B3GNT1* (*B4GAT1*)*, GMPPB, DOLK*, *DPM1, DPM2 and DPM3*. Regardless of the causative gene, α-DGP is characterized by abnormal glycosylation of α-DG, indicating that the disease is associated with defects in the glycosylation pathway for α-DG. α-DG is a cell surface receptor for matrix and synaptic proteins such as laminins, agrin, perlecan, neurexin, and pikachurin^22^,^23^. A unique *O*-mannosyl glycosylation is required for the ligand-binding activity of α-DG, and abnormal glycosylation leads to reduced ligand-binding activity^24^,^25^. α-DG also interacts with a transmembrane β-DG, which in turn binds to intracellular dystrophin^23^. Thus, proper glycosylation of α-DG is necessary for the connection between the basement membrane and cytoskeleton. Disruption of this linkage is thought to cause myofibre membrane weakness, leading to disease-predisposing muscle cell necrosis^26^. Although myofibres can regenerate after necrosis, it has been shown that muscle regeneration activity is impaired in α-DGP^27^. Thus, α-DG glycosylation is important for maintenance of skeletal muscle viability and defects in this process underlie the pathogenesis of α-DGP.

α-DGP includes Fukuyama congenital muscular dystrophy (FCMD), muscle-eye-brain disease (MEB), Walker-Warburg syndrome (WWS), and several types of congenital muscular dystrophies (MDCs) and limb-girdle muscular dystrophies (LGMDs)^1^,^2^. The clinical spectrum of α-DGP is wide; the most severe cases exhibit congenital muscular dystrophy with structural abnormalities in the brain and eyes, whereas the mildest form presents as adult-onset LGMD with no central nervous system involvement^28^,^29^,^30^. In addition, there is no clear genotype-phenotype correlation. Thus, it has been proposed that α-DGPs can be classified into three broad phenotypic groups, MDDG (muscular dystrophy dystroglycanopathy) type A, B and C^29^: MDC with brain/eye abnormalities (A), MDC with milder brain structural abnormalities (B), and LGMD (C). FCMD is the first identified α-DGP^31^ and the second most common childhood muscular dystrophy in Japan^32^,^33^. The causative gene for FCMD is *fukutin*^6^. *LARGE* was identified as the gene responsible for MDC1D^8^. Fukutin and LARGE are involved in a novel phosphodiester-linked modification, namely, post-phosphoryl modification, of *O*-mannnose on α-DG^24^,^34^. Although the exact function of fukutin is unknown, LARGE was recently shown to be a glycosyltransferase that catalyses the formation of a repeating [-3Xyl-α1,3GlcAβ1-] polymer, which is modified on the distal end of the post-phosphoryl moiety^35^. These repeating units likely serve as the ligand-binding domain of α-DG^36^. Interestingly, overexpression of LARGE causes hyperglycosylation of α-DG with increased ligand-binding activity not only in wild-type and *Large*-deficient muscle cells, but also in cells from WWS, MEB, FCMD patients and mouse models^37^. This finding inspired a novel therapeutic strategy based on the unique activity of LARGE—modulation of LARGE activity can be a versatile treatment for α-DGP, regardless of the causative gene.

After this breakthrough finding, several reports showed that overexpression of LARGE in mice induced hyperglycosylation of α-DG in skeletal muscle of α-DGP mouse models such as *POMGnT1*- and *FKRP*-deficient^38^,^39^. However, LARGE overexpression in cells lacking GTDC2 expression or POMT1 activity did not induce hyperglycosylation of α-DG^9^,^40^. Moreover, some studies have shown the beneficial effects of LARGE overexpression in *POMGnT1*- or *FKRP*-mutant mice^38^,^39^, but others showed a deterioration in *FKRP*- or *fukutin*-mutant mice crossed with LARGE-overexpressing transgenic mice^41^,^42^. Thus, it remains unclear whether LARGE could be a target molecule for α-DGP treatment. We hypothesized that the conditions for LARGE expression such as way of gene delivery, timing of intervention, and target cells may affect α-DG glycosylation and therapeutic consequences. Here, we examined therapeutic benefits of myofibre-selective *Large* gene expression after disease manifestation in *Large*- or *fukutin*-deficient α-DGP mouse models. Our data also showed that fukutin is a prerequisite for LARGE-dependent rescue of α-DG glycosylation.

## Results

### Myofibre-selective expression of *Large* after disease onset restores α-DG glycosylation and ameliorates dystrophic pathology of *Large^myd^* mice

**Figure 1:**
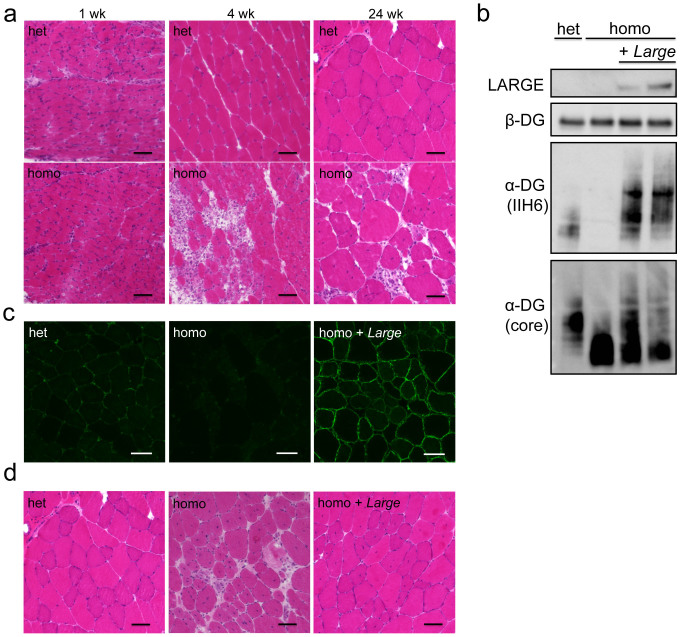
Systemic gene transfer of *Large* into *Large^myd^* mice after onset. (a) Histopathological analysis (H&E staining) of the skeletal muscle of *Large^myd^* (homo) and heterozygous (het) mice at 1, 4, and 24 weeks of age. Bar = 50 μm. (b–d) AAV9-MCK-*Large* was administered to 5-week-old *Large^myd^* mice via the tail vein; after 5 months, the skeletal muscles were harvested and analysed for α-DG glycosylation (b, c) and histology (d). LARGE was detected by western blotting of total lysates (b). β-DG was used as a loading control. DG proteins enriched with WGA-beads were analysed by western blotting to assess α-DG glycosylation (b). The full-length blots with α-DG (IIH6), α-DG (core), LARGE, and β-DG are presented in Supplementary Figure S2a–d, respectively. Immunofluorescence analysis with IIH6 antibody confirmed the increase in α-DG glycosylation (c). H&E staining of the tibialis anterior muscle indicated amelioration of the muscular dystrophic phenotype after treatment with AAV9-MCK-*Large* (d). Het, *Large^myd^* heterozygous controls; homo, untreated *Large^myd^* homozygous mice; and homo + *Large*, *Large^myd^* homozygous mice with AAV9-MCK-*Large* treatment. Bar = 50 μm.

We performed systemic *Large* gene delivery after disease manifestation and myofibre-selective *Large* gene expression in α-DGP mouse models. For this purpose, we constructed recombinant adeno-associated virus (AAV) 9 vectors containing the *Large* cDNA under the myofibre-selective muscle creatine kinase (MCK) promoter (AAV9-MCK-*Large*). We first examined the therapeutic benefits of muscle-selective LARGE expression in *Large*-deficient *Large^myd^* mice. New-born *Large^myd^* mice (1 week old) showed no signs of muscle pathology (Fig. 1a), but at 4 weeks of age, the *Large^myd^* skeletal muscles showed signs of muscular dystrophy such as necrotic and regenerating fibres (Fig. 1a). After 4 months, *Large^myd^* mice showed severe dystrophic pathology in the hind-limb muscles (Fig. 1a). The dystrophic changes include the presence of myofibres with loss of polygonal contour, high population of regenerating fibres with centrally located nuclei, and infiltrations of macrophages and connective tissues. Therefore, we administered intravenous AAV9-MCK-*Large* via the tail vein to 5-week-old *Large^myd^* mice exhibiting dystrophic symptoms, and then analysed α-DG glycosylation status and therapeutic effects after 5 months. Glycosylation status was evaluated by assessing the reactivity of the monoclonal IIH6 antibody, which recognizes properly glycosylated α-DG^25^.

**Figure 2:**
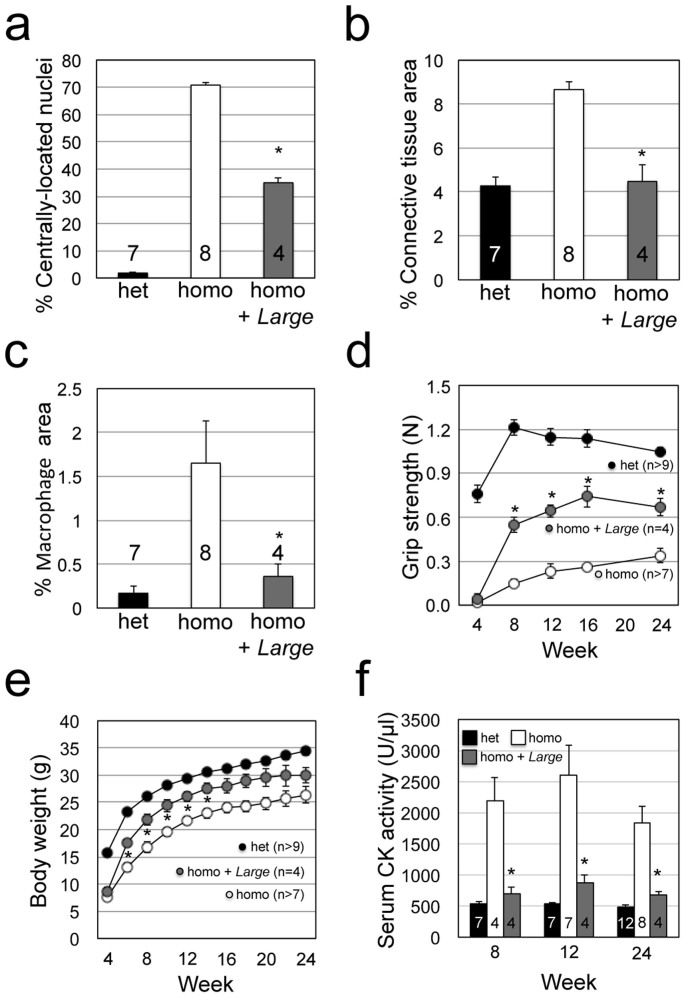
Quantitative analysis of the therapeutic effects of AAV9-MCK-*Large* treatment in *Large^myd^* mice. Amelioration of dystrophic histology after AAV9-MCK-*Large* treatment was evaluated by quantifying muscle fibres with centrally located nuclei (a; *P* = 0.007), measuring infiltration of connective tissue by collagen I-immunofluorescence staining (b; *P* = 0.007) and infiltration of macrophages by F4/80-immunofluorescence staining (c; *P* = 0.011). Therapeutic efficacy over time was evaluated by grip strength (d; *P* = 0.007, 0.006, 0.008, and 0.014 for 8, 12, 16, and 24 weeks), body weight (e; *P* = 0.019, 0.019, 0.024, 0.017, and 0.032 for 6, 8, 10, 12, and 14 weeks), and serum CK activity (f; *P* = 0.021, 0.008, and 0.011 for 8, 12, and 24 weeks). Data shown are mean ± s.e.m. for each group (*n* is indicated in the graph). *P ≤ 0.05 vs. non-treated *Large^myd^* homozygous mice (Mann–Whitney U test). Het, *Large^myd^* heterozygous controls; homo, untreated *Large^myd^* homozygous mice; and homo + *Large*, *Large^myd^* homozygous mice with AAV9-MCK-*Large* treatment.

Western blot analysis confirmed LARGE was overexpressed in AAV-treated *Large^myd^* mice; consequently, the reactivity of IIH6 antibody exceeded even the baseline levels observed in untreated heterozygous animals (Fig. 1b). Immunofluorescence analysis also confirmed increased IIH6-reactivity in the treated *Large^myd^* skeletal muscles (Fig. 1c). Haematoxylin and eosin (H&E) staining of skeletal muscles indicated decreases in the number of necrotic fibres and recovery of the polygonal contour of myofibres in AAV-treated *Large^myd^* versus untreated *Large^myd^* mice (Fig. 1d). The number of muscle fibres with centrally located nuclei as well as infiltration of connective tissues and macrophages were significantly reduced in comparison to the findings obtained for untreated *Large^myd^* mice (Fig. 2a–c). After the AAV-injection, we tracked changes in grip strength, body weight, and serum creatine kinase (CK). Our results showed significant improvements of these parameters even 4 weeks after the injection (Fig. 2d–f). These results demonstrated that myofibre-selective LARGE expression in *Large^myd^* mice via systemic administration ameliorates the dystrophic pathology even if the initial intervention occurs after onset.

### *Large* gene therapy failed to restore glycosylation and ameliorate muscle pathology of *fukutin*-deficient α-DGP models

**Figure 3:**
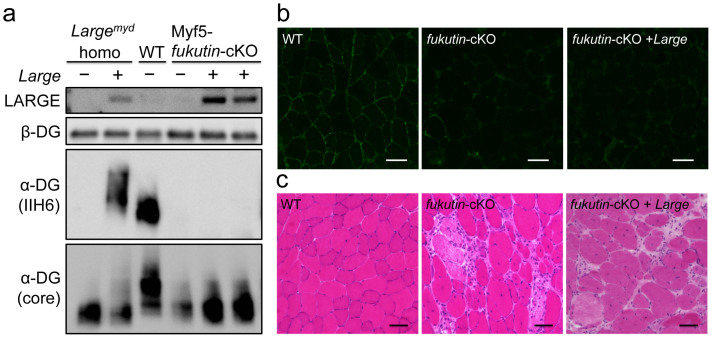
Systemic gene transfer of *Large* into Myf5-*fukutin* cKO mice after onset. AAV9-MCK-*Large* was administered to 4-week-old Myf5-*fukutin* cKO mice via tail vein injection; after 2 months, the skeletal muscles were harvested and analysed for α-DG glycosylation (a, b) and histology (c). Although LARGE was expressed (a), the levels of α-DG glycosylation were unchanged in AAV-treated Myf5-*fukutin*-cKO mice (a, b). H&E staining for the tibialis anterior muscle did not show improvement of the muscular dystrophic phenotype of Myf5-*fukutin*-cKO mice (c). WT, litter control mice (*fukutin^lox/lox^* without cre-transgene); *fukutin*-cKO, untreated Myf5-*fukutin*-cKO mice; and *fukutin* cKO + *Large*, Myf5-*fukutin*-cKO mice with AAV9-MCK-*Large* treatment. Bar = 50 μm. The full-length blots with α-DG (IIH6), α-DG (core), LARGE, and β-DG are presented in Supplementary Figure S2e-h, respectively.

LARGE overexpression increases glycosylation and ligand-binding activity of α-DG in *fukutin*-deficient cells from FCMD patients^37^. We examined whether the muscular dystrophic phenotype of *fukutin*-deficient mice can be improved by LARGE overexpression in vivo. We used muscle precursor cell (MPC)-selective *fukutin*-deficient conditional knock-out (cKO) mice as a *fukutin*-deficient model (Myf5-*fukutin*-cKO mice)^27^. Myf5-*fukutin*-cKO mice showed loss of IIH6-positive glycosylation of α-DG in the skeletal muscles at birth^27^. The dystrophic pathology begins around 4 weeks of age and becomes severe at 12 weeks^27^. We administered intravenous AAV9-MCK-*Large* into 4-week-old Myf5-*fukutin*-cKO mice via the tail vein, and then analysed the glycosylation status of α-DG and therapeutic efficacy after 2 months. Interestingly, although we observed expression of LARGE protein in the AAV-treated Myf5-*fukutin*-cKO skeletal muscles, IIH6-positive α-DG was hardly produced in AAV-treated Myf5*-fukutin* cKO mice (Fig. 3a). Immunofluorescence staining also confirmed failure to restore IIH6-positive glycosylation of α-DG by AAV-treatment in Myf5-*fukutin*-cKO mice (Fig. 3b). H&E staining of skeletal muscles and quantitative muscle pathology showed no significant improvement with AAV treatment (Fig. 3c and Fig. S1a–c). In addition, we found no evidence to support improvements in grip strength, body weight, and serum CK activity after AAV treatment (Fig. S1d–f). These data indicate that the failure to restore α-DG glycosylation in Myf5-*fukutin*-cKO mice is associated with failure of LARGE therapeutic efficacy.

**Figure 4:**
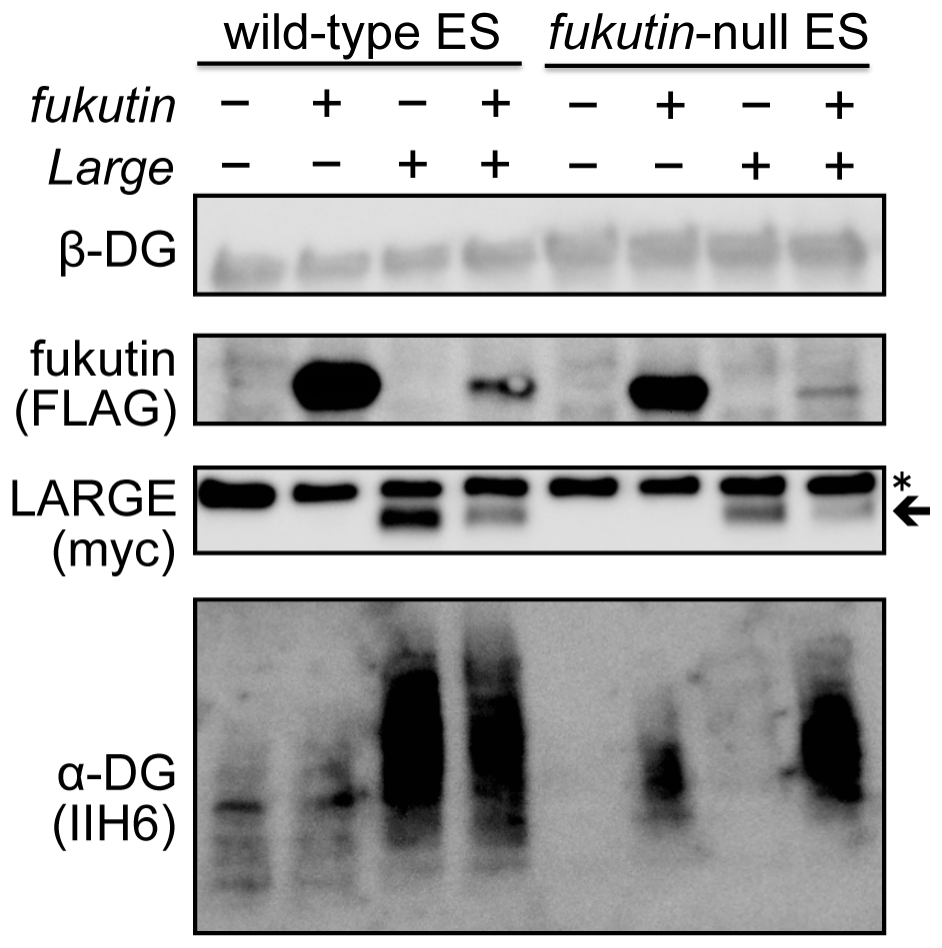
α-DG glycosylation in *fukutin*-null ES cells after fukutin or LARGE expression. Fukutin (FLAG-tagged) and/or LARGE (myc-tagged) were expressed in wild-type or *fukutin*-null mouse ES cells and α-DG glycosylation status was analysed by western blotting. Exogenous LARGE expression produced highly glycosylated α-DG in wild-type but not *fukutin*-null ES cells, indicated by IIH6 staining. Co-transfection of *fukutin* and *Large* yielded LARGE-dependent glycosylation of α-DG in *fukutin*-null ES cells. Arrow and asterisk indicate LARGE protein and non-specific signals, respectively. The full-length blots with α-DG (IIH6), LARGE (myc), fukutin (FLAG), and β-DG are presented in Supplementary Figure S2i-l, respectively.

The amount of LARGE protein expressed in the AAV-treated Myf5-*fukutin*-cKO mice was comparable to that in AAV-treated *Large^myd^* (Fig. 3a). α-DG glycosylation was recovered in *Large^myd^* skeletal muscle after AAV9-MCK-*Large* treatment, suggesting something other than protein expression levels is responsible for the failure of glycosylation recovery in Myf5-*fukutin*-cKO mice. We hypothesized that complete loss of fukutin caused failure to build the part of post-phosphoryl moiety, which may be required for LARGE-dependent glycosylation; therefore, even excess LARGE protein could not form the [-3Xyl-α1,3GlcAβ1-] polymer on α-DG. To test this hypothesis, we expressed LARGE in *fukutin*-null embryonic stem (ES) cells. Transfection of the *fukutin* cDNA restored IIH6 reactivity in *fukutin*-null ES cells, but transfection of the *Large* cDNA failed to restore α-DG glycosylation, although LARGE expression in wild-type ES cells produced strong IIH6-reactivity (Fig. 4). When the *fukutin* and *Large* cDNAs were co-transfected into *fukutin*-null ES cells, we observed increases in IIH6-reactivity in comparison to *fukutin* singly transfected cells although expression levels of both fukutin and LARGE were much lower than they were in each single transfection (Fig. 4, lanes 6–8). These data show that fukutin-dependent modification is a prerequisite for LARGE-dependent formation of [-3Xyl-α1,3GlcAβ1-] repeating units.

## Discussion

It has been widely recognized that LARGE possesses the great potential to rescue glycosylation defects of α-DG regardless of causative genes for α-DGP, however, therapeutic benefits of using LARGE activity is controversial. To assess the feasibility of LARGE-utilizing therapy, in this study we explored the conditions needed for successful *Large* gene therapy. We demonstrated for the first time that AAV-mediated *Large* gene expression targeted to myofibres is therapeutically beneficial in a *Large*-deficient α-DGP model even when the intervention is performed after disease manifestation. On the other hand, the same strategy failed to restore α-DG glycosylation in *fukutin*-deficient skeletal muscles and ES cells, suggesting that fukutin is required for LARGE-utilizing therapeutic strategy.

Several reports have shown that AAV-mediated LARGE expression, which was driven by the chicken β-actin (CB) promoter, restored glycosylation of α-DG in *Large^myd^* mice^38^,^39^. However, expression of exogenous genes by non-selective promoter such as the CB promoter in various non-muscle cells may be detrimental. For example, because glycosylation levels of α-DG and the expression levels of LARGE change during muscle differentiation and regeneration^36^, excess glycosylation may disturb cellular homeostasis and tissue regeneration. In fact, LARGE overexpression in C2C12 myoblasts impairs differentiation^42^. Furthermore, there may be situations or cells in which glycosylation is physiologically unnecessary even in wild-type tissues^34^. Although AAV-mediated *Large* gene transfer using the CB promoter rescued the muscular dystrophic phenotype in *Large^myd^* mice^38^,^39^, this study was designed to introduce viral vectors into new-born pups; thus, it is likely that α-DG restores proper glycosylation before disease manifestation, preventing disease-causing myofibre necrosis. However, in humans, clinical and genetic diagnoses of α-DGP are made after disease manifestation, making gene delivery in new-borns unfeasible. We demonstrated that myofibre-selective expression of LARGE ameliorated the dystrophic phenotype of *Large^myd^* mice, and the intervention even after disease manifestation was effective by using systemic AAV-mediated gene delivery. The effect continues at least for 5 months after the intervention, the longest period observed in gene therapy studies of α-DGP mouse models. Myofibre-selective rescues of fukutin or LARGE expression by AAV gene transfer or crossing with transgenic mice improved the dystrophic phenotypes of Myf5-*fukutin*-cKO or *Large^myd^* mice^27^,^43^, supporting the validity of myofibre-selective gene rescue for treatment of α-DGP. We propose that myofibre-targeting rescue/reinforcement of glycosylation is an effective treatment for α-DGP.

We also examined the potential of LARGE as a therapeutic target for *fukutin*-deficient α-DGP; however, our data showed that LARGE expression failed to restore α-DG glycosylation in *fukutin*-null myofibres and ES cells. LARGE synthesises [-3Xyl-α1,3GlcAβ1-] repeating units in the terminal end of the post-phosphoryl moiety, which is modified on phospho-mannose in the Core M3 structure, GalNAc-GlcNAc-Man(P)-O^24^,^35^,^44^. The activities of POMT and GTDC2 are required for Core M3 synthesis and fukutin mediates formation of the post-phosphoryl modification^24^,^34^,^44^. In cells lacking POMT activity or GTDC2 expression, LARGE overexpression failed to induce hyperglycosylation or restore glycosylation of α-DG^9^,^40^. Thus, even when overexpressed, LARGE requires the Core M3 structure and fukutin-dependent modification in order to form the [-3Xyl-α1,3GlcAβ1-] repeating units. However, several reports have shown LARGE-dependent glycosylation recovery in genetically distinct α-DGP models^38^,^39^, which seems contradictory but can be explained by the presence of residual glycosylation of α-DG. Presumably, α-DG is hypoglycosylated in such cells, but there might be a small amount of normally glycosylated α-DG species produced by residual activity of the mutant gene products. In fact, the *fukutin*-knock-in and *POMGnT1*-KO mice showed slight reactivity against IIH6 antibody, indicating the presence of normally glycosylated α-DG^45^,^46^. In such cases, LARGE can reinforce [-3Xyl-α1,3GlcAβ1-] repeats on the small amount of residual Core M3 structure. Importantly, many mild cases of FCMD patients show the presence of a small fraction of normally glycosylated α-DG^30^. Thus, the concept of LARGE modulation therapy remains attractive for a wide range of mild cases of α-DGP such as MDDG type C.

An important issue when considering therapeutic applications is that α-DGP is accompanied by central nervous system abnormalities including severe defects in structural development and mental retardation^28^,^29^. Although the AAV9 vector delivers genes to central nervous tissues^47^, there are many obstacles to treatment for developing central nervous tissues. For example, the structural abnormalities in the central nervous system occur during fetal developing stage^48^,^49^, and such abnormalities are thought to be irreversible after birth. Moreover, fetal therapy also contains many concerns such as ethical, legal, technical, and clinical safety issues. However, improvement of muscle functions via muscle-targeting strategies will improve patients' activities of daily living and caregivers' burdens, and may have an impact on patient mental development. We propose that gene therapy is an effective approach to α-DGP even after disease progression, and that LARGE therapy may be applied to mild cases of α-DGP. Our study will provide a new direction for therapeutic approached to α-DGP.

## Methods

### Animals

*Large*-deficient *Large*^myd^ mice were from Jackson Laboratories. Generation of muscle precursor cell (MPC)-selective *fukutin* conditional knock-out (cKO) mice (Myf5-*fukutin*-cKO mice) was described previously^27^. All animal procedures were approved by the Animal Care and Use Committee of Kobe University Graduate School of Medicine (P120202-R2) in accordance with the guidelines of the Ministry of Education, Culture, Sports, Science and Technology (MEXT) and the Japan Society for the Promotion of Science (JSPS). The animals were housed in cages (2–4 mice per cage) with wood-chip bedding in an environmentally controlled room (25°C, 12 h light-dark cycle) and provided food and water *ad libitum* at the animal facility of Kobe University Graduate School of Medicine. Well-trained and skilled researchers and experimental technicians, who have knowledge of methods to prevent unnecessary and excessive pain, handled the animals and performed the experiments. Euthanisation was done by cervical dislocation. At sacrifice, the muscles were harvested and snap-frozen in liquid nitrogen (for biochemistry) or in liquid-nitrogen-cooled isopentane (for immunofluorescence and histology). The number and ages of the animals used in each experiment are indicated in the Figure legends and graphs.

### Adeno-associated viral gene transfer and evaluation of therapeutic efficacy

To generate a *Large*-encoding AAV9 vector, the complete open reading frame of the mouse *Large* gene was cloned into pAAV-IRES-hrGFP^50^. The MCK promoter was subcloned from AAV-MCKLacZ^51^. The recombinant *Large*-encoding AAV9 vector (AAV9-MCK-*Large*) was produced as described^50^. The AAV9-MCK-*Large* viral vectors (5 × 10^11^ vector genome) were injected into *Large^myd^* (n = 4) and Myf5-*fukutin* cKO (n = 6) mice via tail vein at 4–5 weeks of age. Before the injection, body weight, grip strength, and serum CK activity were measured. These clinical parameters (body weight, grip strength, and serum CK activity) were continuously measured until the AAV-treated mice were sacrificed for histological evaluation (the time points of the clinical tests were shown in the figures). The H&E and immunofluorescence images shown in the figures are representative of the AAV-treated and non-treated mice.

Quantitative evaluation of muscle pathology was performed by assessing the number of myofibres with centrally located nuclei at least 1,000 fibres. Macrophage and connective tissue infiltration was quantified by analysing the immunofluorescence signals of F4/80-positive and collagen I-positive areas with Image J software. Serum CK activity was measured with the CPK kit (WAKO). Grip strength was measured for 10 consecutive trials for each mouse using a strength meter (Ohara Ika Sangyo Co. Ltd., Tokyo); 20% of the top and bottom values were excluded to obtain the mean. Statistical analysis was performed to determine means and s.e.m.; *P*-values <0.05 were considered significant (Mann–Whitney U test).

### Antibodies

Antibodies for western blotting and immunofluorescence were as follows: mouse monoclonal antibody 8D5 against β-DG (Novocastra); mouse monoclonal antibody IIH6 against α-DG (Millipore); rat monoclonal antibody against mouse F4/80 (BioLegend); rabbit polyclonal antibody against collagen I (AbD Serotec); mouse monoclonal antibody against FLAG tag (Sigma); and rabbit polyclonal antibody against c-Myc tag (Santa Cruz). Rabbit polyclonal antibody against LARGE was raised using recombinant human LARGE protein expressed in *E. coli*. Antisera were purified by Melon gel IgG purification kit (Pierce). Rat monoclonal antibody against the α-DG core protein (3D7) was generated from a α-DG-Fc fusion protein^52^.

### Protein preparation and western blotting

DG was enriched from solubilized skeletal muscle as described^27^. Briefly, skeletal muscles (TA ~30 mg, calf ~100 mg) were solubilised in Tris-buffered saline containing 1% Triton X-100 and protease inhibitors (Nacalai). The solubilised fraction was incubated with wheat germ agglutinin (WGA)-agarose beads (Vector Laboratories) at 4°C for 16 h; DG was eluted with SDS-PAGE loading buffer. To detect LARGE protein expression, total lysates were analysed by western blotting. Proteins were separated in 4–15% linear gradient SDS gels, then transferred to polyvinylidene fluoride (PVDF) membranes (Millipore). Blots were probed with antibodies and developed with horseradish peroxidase (HRP)-enhanced chemiluminescence reagent (Supersignal West Pico, Pierce; or ECL Prime, GE Healthcare).

### Histology and immunofluorescence analysis

For H&E staining, cryosections (7 μm) were stained for 2 min in haematoxylin, 1 min in eosin, and dehydrated with ethanol and xylene. IIH6-immunofluorescence analysis was performed after treating the with cold ethanol/acetic acid (1:1) for 1 min, blocking with 5% goat serum in MOM Mouse Ig Blocking Reagent (Vector Laboratories) at room temperature for 1 h, and incubation with primary antibodies diluted in MOM Diluent (Vector Laboratories) overnight at 4°C. For F4/80- and collagen I-immunofluorescence, sections were blocked with 3% bovine serum albumin (BSA) in phosphate-buffered saline (PBS) at room temperature for 1 h, and then with primary antibodies diluted in 1% BSA overnight at 4°C. The slides were washed with PBS and incubated with Alexa Fluor 488-conjugated or Alexa Fluor 555-conjugated secondary antibodies (Molecular Probes) at room temperature for 30 min. Permount (Fisher Scientific) and TISSU MOUNT (Shiraimatsu Kikai) were used for H&E staining and immunofluorescence, respectively. Sections were observed by fluorescence microscopy (Leica DMR, Leica Microsystems).

### ES cell culture

ES cells were cultured in DMEM with 20% heat-inactivated foetal bovine serum, 100 μM 2-mercaptoethanol, 1 mM non-essential amino acids (Gibco), 2 mM L-glutamine (Nacalai), and 10^3^ U/mL leukaemia inhibitory factor (Millipore). Targeted disruptions of the *fukutin* gene in ES cells have been described previously^53^. Transfection was performed with Lipofectamine LTX reagents (Invitrogen) according to manufacturer protocols. After 48 h transfection, cells were lysed with Tris-buffered saline containing 1% Triton X-100 and protease inhibitors (Nacalai). DG proteins were enriched with WGA-beads and analysed by western blotting.

## Author Contributions

Y.O., M.K., T.O., K.K., S.T. and T.T. conceived and designed the research. Y.O., M.K. and C.I. performed experiments and analysed the data. C.Y., K.K., T.C. and T.O. constructed the AAV vectors. Y.O., M.K. and T.T. wrote the paper. All authors reviewed the manuscript.

## Supplementary Material

Supplementary Information Supplementary Figures
