# Supplementary material for: Fukutin is prerequisite to ameliorate muscular dystrophic phenotype by myofiber-selective LARGE expression
Source: Sci Rep. 2015 Feb 9;5:8316. doi: 10.1038/srep08316 (PMC4321163; doi:10.1038/srep08316)
Supplement: Supplementary Information — Supplementary Figures [file srep08316-s1.doc]

**Fukutin is prerequisite to ameliorate muscular dystrophic phenotype by myofiber-selective LARGE expression**

Yoshihisa Ohtsuka^1^, Motoi Kanagawa^1^, Chih-Chieh Yu^1^, Chiyomi Ito^1^, Tomoko Chiyo^2^, Kazuhiro Kobayashi^1^, Takashi Okada^2^, Shin’ichi Takeda^2^, and Tatsushi Toda^1*^


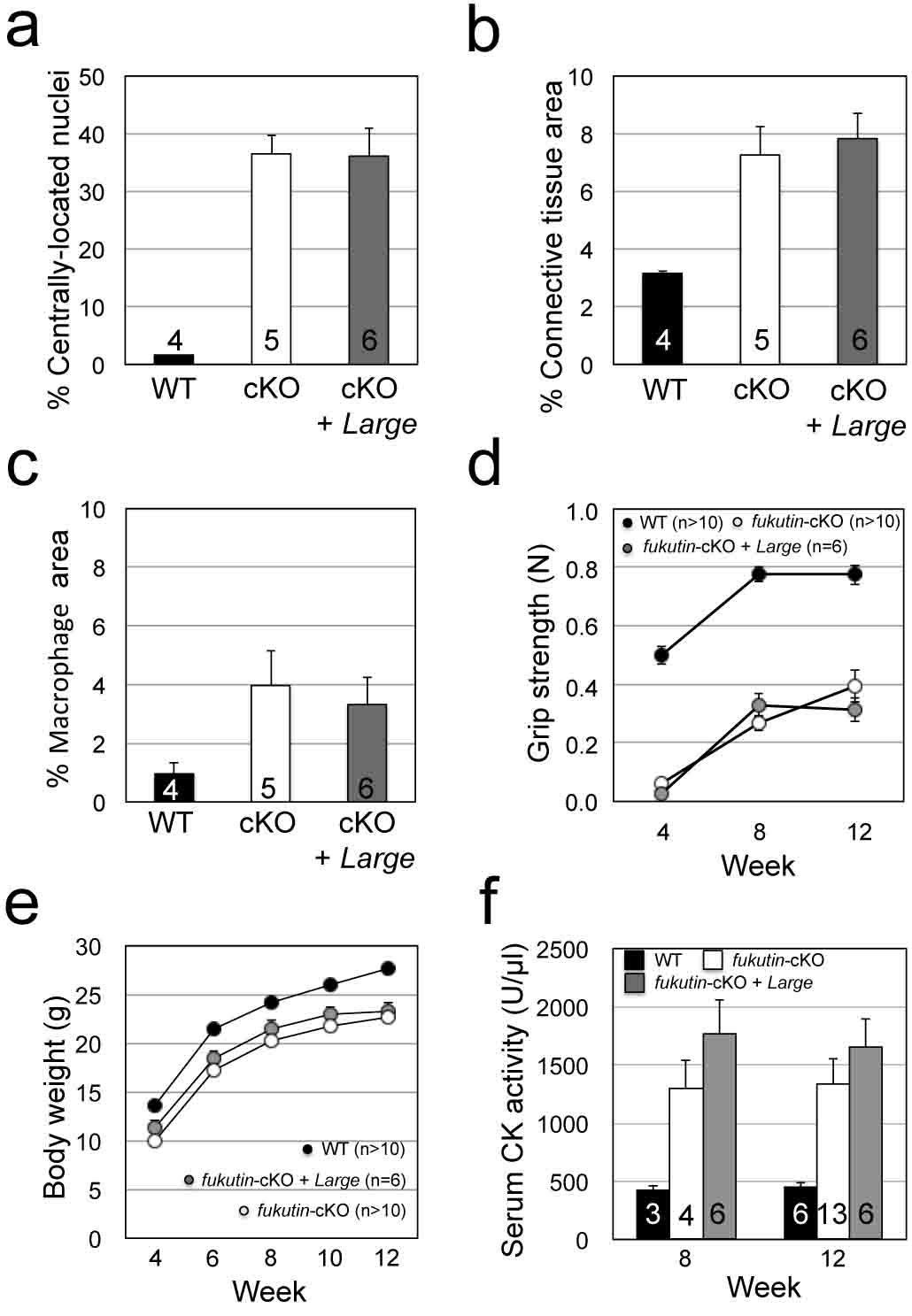


**Supplementary Figure S1. Quantitative analysis of the therapeutic effects of AAV9-MCK-*Large* treatment in Myf5-*fukutin* cKO mice**

Histopathology of Myf5-*fukutin* cKO skeletal muscle (tibialis anterior) after AAV9-MCK-*Large* treatment was evaluated by quantifying the number of muscle fibres with centrally-located nuclei (a), infiltration of connective tissue (b), and infiltration of macrophages (c). Therapeutic efficacy over time was evaluated by grip strength (d), body weight (e), and serum CK activity (f). Data shown are mean ± s.e.m. for each group (*n* is indicated in the graph). WT, litter control mice (*fukutin^lox/lox^* without cre-transgene); *fukutin*-cKO, untreated Myf5-*fukutin*-cKO mice; and *fukutin* cKO + *Large*, Myf5-*fukutin*-cKO mice with AAV9-MCK-*Large* treatment. **P* ≤ 0.05 vs. the non-treated cKO mice (Mann–Whitney U test).


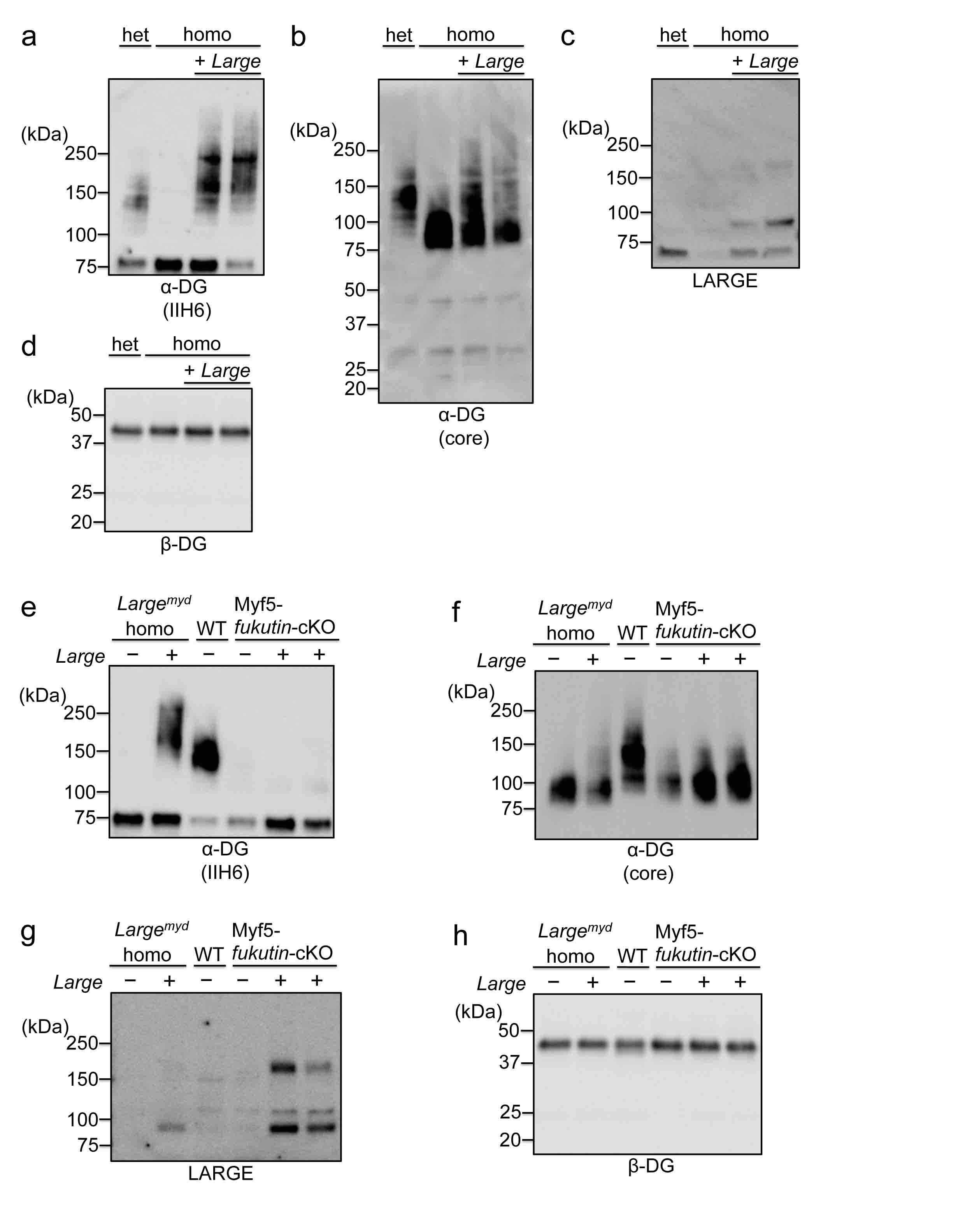

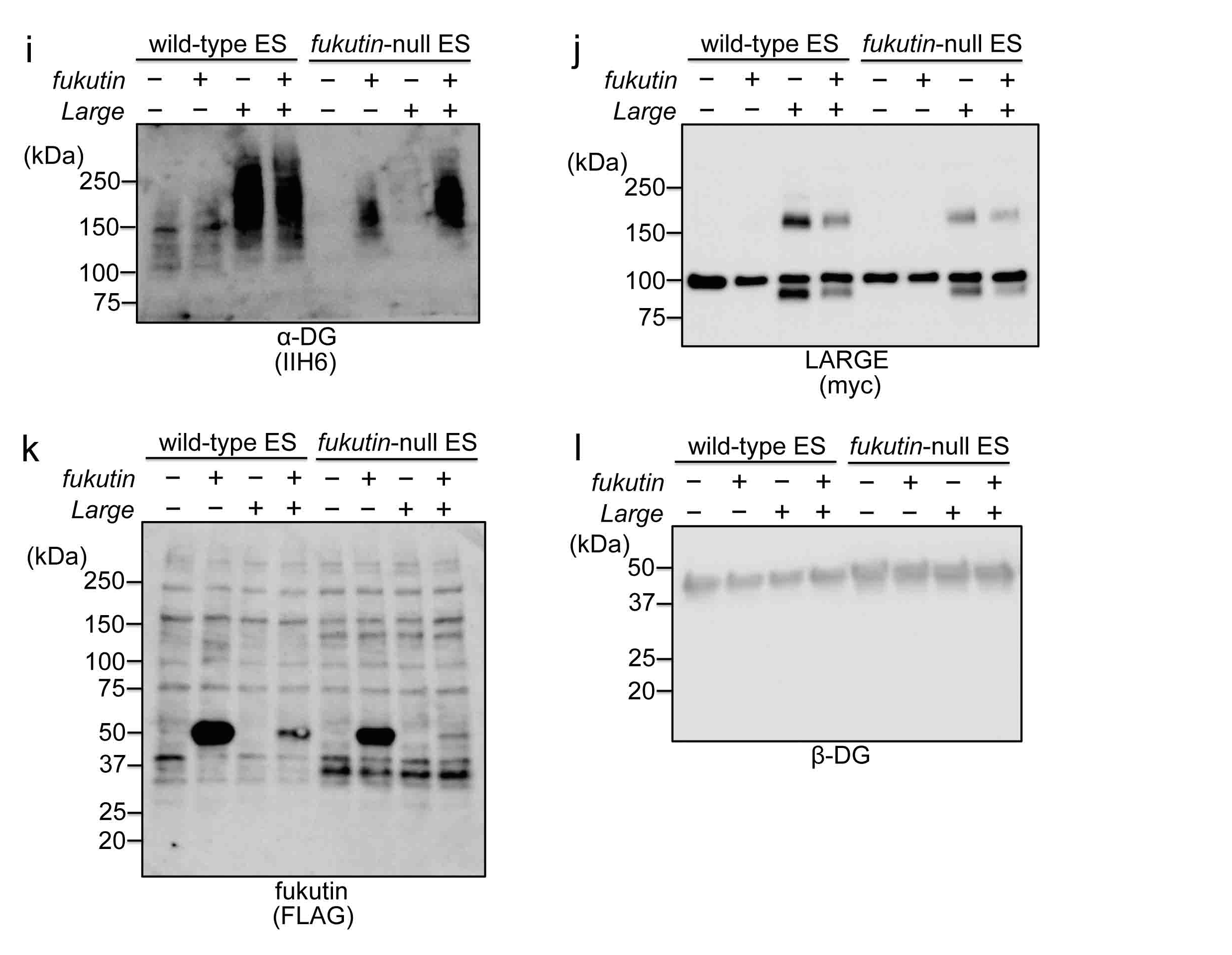


**Supplementary Figure S2. Full-length blots**

The regions of intrest are highlighted in main Figs: (a, b, c, and d) Fig. 1b; (e, f, g, and h) Fig. 3a; and (i, j, k, and l) Fig. 4.
